# Indoor Air Quality in Brazilian Universities

**DOI:** 10.3390/ijerph110707081

**Published:** 2014-07-11

**Authors:** Sonia R. Jurado, Antônia D. P. Bankoff, Andrea Sanchez

**Affiliations:** Federal University of Mato Grosso do Sul. Avenue Ranulpho Marquês Leal, 3484, Três Lagoas 79620-080, Mato Grosso do Sul, Brazil; E-Mails: dallabankoff@bol.com.br (A.D.P.B.); andrea-ufms@hotmail.com (A.S.)

**Keywords:** classroom air quality, biocontaminants, universities, student performance

## Abstract

This study evaluated the indoor air quality in Brazilian universities by comparing thirty air-conditioned (AC) (*n* = 15) and naturally ventilated (NV) (*n* = 15) classrooms. The parameters of interest were indoor carbon dioxide (CO_2_), temperature, relative humidity (RH), wind speed, viable mold, and airborne dust levels. The NV rooms had larger concentration of mold than the AC rooms (1001.30 ± 125.16 and 367.00 ± 88.13 cfu/m^3^, respectively). The average indoor airborne dust concentration exceeded the Brazilian standards (<80 μg/m^3^) in both NV and AC classrooms. The levels of CO_2_ in the AC rooms were significantly different from the NV rooms (1433.62 ± 252.80 and 520.12 ± 37.25 ppm, respectively). The indoor air quality in Brazilian university classrooms affects the health of students. Therefore, indoor air pollution needs to be considered as an important public health problem.

## 1. Introduction

Indoor air quality has caught the attention of scientists and the general public in recent years. There is a growing concern in many countries about air quality in schools. Most epidemiological studies in school settings have been performed in northern Europe; however, other case studies are available for other regions [1,2].

A study performed in Australian schools showed indoor pollution levels greater than outdoor levels [3]; because people spend more than 90% of their time indoors, good indoor air quality is very important. Air pollutants produced by outdoor sources affect the environment and our health. Air quality in classrooms is of special concern for students, particularly those sensitive to poor air quality. These problems can be subtle and do not always produce easily recognizable impacts on the health and welfare of populations [4]. The investigation of air quality in classrooms contributes to not only assessing indoor pollution levels, but also implementing corrective measures to improve air quality.

Indoor air pollution results from the combination of effects from physical, chemical, and biological factors and inadequate ventilation in the environment. Indoor sources of air pollution are heating, ventilation and air conditioning (HVAC) systems, building equipment, furnishings, and human activities. The major outdoor air pollution sources are traffic, industrial, and construction activities [5].

Good ventilation systems control temperature and humidity, provide thermal comfort, distribute adequate amounts of air, and remove pollutants. An air-conditioned room does not imply exclusion of outdoor air pollutants.

Thermal comfort is one of the primary elements determining the quality of the indoor environment, and it is essential to the health of those who must routinely stay indoors over extended periods of time [2]. Environmental comfort is primarily determined by the design of the building, the activity exercised by its occupants, specifically in relation to thermal comfort, and the clothes they wear.

Indoor air pollution in classrooms may increase the chance of short- and long-term health problems for students and staff reducing teachers’ productivity student’s learning possibilities [6,7,8,9]. 

Prolonged exposure to polluted indoor environments may cause various symptoms such as headaches, dizziness, nausea, fatigue, and dry skin. In 1982, a group of experts within the World Health Organization (WHO) described this multitude of symptoms and perceptions as sick building syndrome (SBS). A building can be diagnosed as sick if 20% or more of its occupants exhibit one or more of the above mentioned symptoms for two weeks and such symptoms disappear when leaving the building [10]. 

Some studies were conducted in different indoor environments; however, indoor air quality in the classrooms of Brazilian universities has not been investigated. This study characterized the air pollution level in classrooms from five universities in Brazil, and compared the measured concentrations between NV and AC classrooms in order to recommend ways to reduce pollution levels in classrooms.

## 2. Experimental Section

### 2.1. Site Description and Study Population

The study was conducted between 1 February and 30 March of 2006 during summer; samples were collected between 9 and 11 am, from Monday to Friday, in five Brazilian universities, in the State of Mato Grosso do Sul, including the cities of Três Lagoas and Campo Grande. The universities were located in the suburbs (*n* = 4) or central area (*n* = 1) (Table 1). The institutions’ deans or administrators formally agreed to participate in this study by signing an informed consent. They were informed about the study’s methodology and potential benefits. Six classrooms with similar characteristics such as level in the university building, surface, volume, number of windows, windows structure, number of occupants, activities, and internal covering including flooring, wall, and ceiling were chosen in each university. Therefore, thirty classrooms were studied (*n* = 15, NV rooms, and *n* = 15, AC rooms) and data was collected when students were in the rooms. A total of 1290 samples for each studied parameter were collected during this study, 43 samples per classroom.

**Table 1:** Characteristics of the studied universities.

Monitored Sites	Description
University A	Located in a suburban area
	Surrounded by residences and industries
	Use of a blackboard and chalk
	Built in the 70s
	Presence of vegetation nearby
University B	Located in a suburban area
	Surrounded by residences and buildings
	Use of a blackboard and chalk
	Built in 2000
	No presence of vegetation nearby
University C	Located in a suburban area
	Surrounded by residences and buildings
	Use of blackboard with chalk
	Built in the 70s
	No presence of vegetation nearby
University D	Located in a central area
	Surrounded by residences and buildings
	Use of a blackboard and chalk
	Built in the 70s
	Presence of vegetation nearby
University E	Located in a suburban area
	Surrounded by residences and buildings
	Use of a blackboard and chalk
	Built in the 90s
	No presence of vegetation nearby

A total of 802 students (568 women and 234 men) with an average age of 27 years were recruited. The average number of students in the NV and AC rooms was 29 and 24, respectively. Each participant was informed that all personal data would be confidentially treated; all participants agree on their participation by signing a volunteer informed consent.. Study participants were asked to report eye, nose, and throat irritation, headaches, concentration problems, fatigue, lethargy, and absenteeism due to respiratory diseases xperienced within the preceding four weeks. The response categories of these symptoms were ‘no’ or ‘yes’. The questionnaire also included questions about smoking habits, daily exposure to tobacco, and the presence of pets at home. 

The thermal sensation reporting scores was as follows: too hot: +3; hot: +2; mildly hot: +1; well, nor hot or cold: 0; mildly cold: −1; cold: −2; too cold: −3. Participants who voted +3, +2, −3, and –2 on this seven point perception scale were considered thermally dissatisfied. Participants who voted +1 and −1 were considered thermally satisfied, according to the ASHRAE Scale [11].

### 2.2. Environmental and Occupancy Parameters

The studied parameters and pollutants were indoor carbon dioxide (CO_2_), temperature, relative humidity (RH), wind speed, airborne dust or particulate matter with diameters less than 10 μm (PM10), and indoor and outdoor viable fungi. All measurements were taken in duplicate. The indoor/outdoor fungi concentration ratio (I/O) was calculated; ***I*** is the amount of fungi in the indoor environment and **O** is the amount of fungi in the outdoor environment.

Sampling devices were placed at 1.5 m above ground level in both indoor and outdoor locations. CO_2_ concentrations were measured using a Testo Digital 535 non-dispersive infrared sensor (Testo Inc., Sparta, NJ, USA). Temperature and relative humidity were measured using the Testo Digital 605-H1. Wind speed was measured using the Testo Digital 405-V1. 

Fungi were sampled using the Andersen sampler, MAS-100 model (Merck, Darmstadt, Germany), operating at the fixed volume of 250 L. Sabouraud’s Dextrose agar (SDA) at pH 5.6 was used for air and swab sampling. Plates were incubated at 22 °C and 90% relative humidity (RH) for up to 14 days. After incubation, colonies were counted with the aid of a stereoscopic microscope. The number of colony-forming units (cfu) of fungi in each sample was calculated as cfu/m^3^. Airborne dust levels (PM_10_) were measured using a GILIAN TM instrument (model BDX II, Sensidyne, Clearwater, FL, USA). The levels of all measured parameters from the two ventilation systems (NV and AC rooms) were compared.

### 2.3. Statistics

The non-parametric Mann-Whitney test was used for comparisons of all studied parameters. The outcomes of interest (indoor fungal concentration, indoor airborne dust, indoor CO_2_ levels, pets at home, and daily exposure to tobacco) were tested for linear correlation with the buildings-related symptoms (Pearson r). *p* < 0.05 was considered as statistically significant.

## 3. Results

A summary of the statistical analyses of the indoor climate variables is shown in Table 2. 

**Table 2:** Indoor environmental quality parameters evaluated in classrooms.

Pollutant/Parameter	NV	AC	Brazilian Recommended Values
Room temperature (°C)	28.87 ± 0.57	25.87 ± 0.65 *	23–26 (in summer)
Relative air humidity (%)	65.00 ± 2.13	56.21 ± 1.70 *	40–65 (in summer)
Wind speed (m/s)	0.14 ± 0.01	0.09 ± 0.01 *	≤0.25
CO_2_ (ppm)	520.12 ± 37.25	1433.62 ± 252.80 *	≤1000
Airborne dust (μg/m^3^)	215.12 ± 68.20	659.22 ± 102.80 *	≤80
Viable fungi (cfu/m^3^)	1001.30 ± 125.16	367.00 ± 88.13 *	≤750
I/O	1.08 ± 0.21	0.45 ± 0.09 *	≥1.5

I/O: indoor/outdoor fungi concentration ratio. Values are expressed as mean ± standard error. * *p* < 0.05.

Relative air humidity and indoor temperature values were within acceptable ranges in the majority of AC rooms. This was not observed in the NV classrooms, which showed mean temperatures above 26 °C (Table 2). The indoor RH recommended by the national guidelines must be between 40% and 65%. The indoor temperature suggested by the Brazilian standards must be between 23 and 26 °C in the summer, and 20 and 22 °C in the winter [12]. The indoor wind speed detected values were within the Brazilian standards in both types of rooms (Table 2).

Carbon dioxide (CO_2_) concentrations can be considered as an indicator of ventilation rate. The results showed that the detected CO_2_ concentrations varied among classrooms and according to ventilation type, window operation, and room occupation rate. The highest CO_2_ concentrations (frequently exceeding 1000 ppm) occurred in the AC rooms (Table 2). The values of CO_2_ concentration detected in the NV rooms were within the Brazilian guidelines. 

The detected airborne dust concentration were significantly higher in the AC rooms (659.22; ranging from 181.82 to 1091.91 μg/m^3^) than in the NV rooms (215.12μg/m^3^; ranging from 18.45 to 5435.46 μg/m^3^) (Table 2). The recommended Brazilian value for airborne dust concentration is ≤80 μg/m^3^ [12]. This suggests that dust within the NV rooms was of environmental origin. In the AC rooms, outdoor dust input and lack of cleanliness of air conditioner filters were observed.

The NV rooms tended to have higher fungal concentration than the AC rooms (1001.30 *vs.* 367.00, respectively, *p* < 0.05) (Table 2). Opening windows during part of the day, increased outdoor temperature, and outdoor fungal concentrations are related to the increased indoor fungal concentrations in the NV rooms. Indoor biocontamination was assessed using the indoor/outdoor (I/O) fungi concentration ratio. I/O ratios above 1.5 reveal indoor air pollution due to insufficient ventilation, inappropriate cleaning practices, or outdoor contamination [12]. Both NV and AC rooms showed I/O < 1.5.

Almost 68% of the participants had pets at home, 27% reported daily exposure of tobacco, and 8% were smokers. Daily exposure to tobacco was positively and significantly associated with general and specific symptoms of SBS. There were no significant differences in building-related symptoms and absenteeism between occupants of the NV and AC rooms (Table 3). Among the study participants, 45% were allergic to dust, drugs, chemicals, and other allergens. 

**Table 3:** Symptoms and absenteeism in students from NV and AC classrooms.

Type	Naturally Ventilated (*n* = 444)	Air-conditioned (*n* = 358)
*Specific symptoms*		
Eye irritation (%)	26.0	24.0
Nasal irritation (%)	28.0	32.0
Throat irritation (%)	22.0	16.0
*General symptoms*		
Headache (%)	18.0	20.0
Difficulties to concentrate (%)	23.0	25.0
Fatigue (%)	28.0	26.0
Lethargy (%)	23.0	17.0
*Absenteeism* (%)	12.0	9.0

AC room participants had statistically significant better scores compared to those from the NV rooms (7.00 ± 0.15 *vs.* 6.40 ± 0.13). Academic performance was negatively associated with higher room temperatures.

Pets at home were significantly associated with all specific and general symptoms of SBS. The concentration of fungi was positively and significantly correlated with the occurrence of headaches, concentration problems, and fatigue in students from the NV rooms. The CO_2_ levels were positively and significantly correlated with all general and specific symptoms of SBS, except lethargy, in the students from the AC rooms. The levels of airborne dust were positively and significantly correlated with throat irritation in the students from the AC rooms (Table 4).

**Table 4:** Building-related symptoms according to the levels of airborne fungi, airborne dust (AD), and carbon dioxide in NV and AC rooms.

Symptoms of SBS	*Fungi*		*CO_2_*		*AD*	
	NV	AC	NV	AC	NV	AC
*Specific symptoms*						
Eye irritation	0.21	0.34	0.11	0.59 ^#^	0.12	0.24
Nasal irritation	0.33	0.36	0.20	0.52 ^†^	0.10	0.18
Throat irritation	0.21	0.43	0.27	0.56 ^†^	0.15	0.47^†^
*General symptoms*						
Headache	0.56 *	0.36	0.11	0.57 *	0.17	0.08
Difficulties in concentration	0.61 *	0.23	0.13	0.61 *	0.20	0.07
Fatigue	0.71	0.32	0.22	0.68 *	0.18	0.11
Lethargy	0.59	0.15	0.19	0.68	0.48	0.49

* *p* < 0.001; ^#^ *p* < 0.01; ^†^ *p* < 0.05.

The thermal sensation evaluation showed that 24.0% and 32.0% of the students were thermally satisfied in the NV and AC rooms, respectively. The percentages of dissatisfied students were 36% and 13% in the NV and AC rooms, respectively (Table 5). The ISO 7730 suggest that the percentage of dissatisfied people should be lower than 10% in a thermally acceptable environment [11].

**Table 5:** Perception of indoor climate by occupants of classrooms.

ASHRAE Scale	NV	AC
	*n* = 444	*n* = 358
–3 cold	0.0%	1.0%
–2 cool	0.0%	4.0%
–1 slightly cool	3.0%	25.0%
0 neutral	40.0%	55.0%
+1 slightly warm	21.0%	7.0%
+2 warm	29.0%	8.0%
+3 hot	7.0%	0.0%

## 4. Discussion

The NV rooms had higher temperatures than AC rooms. Students from AC rooms were more thermally satisfied than those from the NV rooms. Thermal-comfort perceptions are affected by six thermal variables: air temperature, radiant temperature, relative humidity, air velocity, metabolic rate of occupants in various activities, and clothing [13]. Room occupants clearly perceive temperature changes.

In this study, the students from the AC rooms demonstrated better academic performance than those from the NV rooms. There is good evidence that moderate changes in room temperature, even within the comfort zone, affect students’ abilities to perform mental tasks that require concentration and sentence comprehension [14]. 

The AC and NV rooms showed relative humidity and wind speed within Brazilian standards; however, the values were statistically different between both types of rooms. A comfortable environment condition would be 26 °C and 50%–60% relative humidity [15].

The highest CO_2_ concentrations occurred in the AC rooms due to poor air renovation. Indeed, in poorly ventilated classrooms the CO_2_ concentrations can reach values up to 2600 ppm [16]. Increasing the ventilation rate could also remove accumulated CO_2_, for example, by using ceiling fans and exhaust fans that could increase the exchange between indoor and outdoor air; however, increased ventilation rates could also increase the indoor concentration of outdoor generated pollutants. 

The measured CO_2_ levels in the NV rooms were within the specified Brazilian guidelines. We did not observe an association between CO_2_ levels and decreased academic performance in the participants from the AC rooms. Sheehy, Kamon, and Kiser [17] exposed study participants to 4% and 5% CO_2_ (with 21% and 50% O_2_) for 16 min and found no deterioration in psychomotor performance (simple reaction time, tracking pursuit, and choice response time) or mental tasks (short-term memory and reasoning). Another study exploring the effects of acute CO_2_ exposure (15 min or less) found little evidence of impaired mental performance due to breathing up to 6% CO_2_ concentration [18]. Staish and colleagues [19] conducted an experiment with 22 participants exposed to CO_2_ at 2500 ppm in an office-like chamber where participants completed a computer-based test on decision-making performance. At 2500 ppm, extensive and statistically significant reductions occurred in seven out of the nine scales of decision-making performances. A study carried out by Awbi [20] showed that high CO_2_ levels, up to 4000 ppm, impaired the students’ ability to properly concentrate. Subsequent studies are necessary to compare academic performances in air-conditioned rooms with CO_2_ above 1000 ppm including students at different ages (children, adolescents, and adults). 

However, the highest levels CO_2_ were positively and significantly associated with all general and specific symptoms of SBS, except lethargy. Research [21] revealed a partial correlation between symptoms of headaches, dizziness, heavy headedness, tiredness, and difficulty to concentrate among occupants in rooms with high CO_2_ concentrations. Another study with 1607 adolescents found that the elevated CO_2_ concentrations in classrooms were associated with wheeze and cough [22]. Nkwocha and Egejuru [23] reported that some symptoms of high CO_2_ concentration exposure cause prevalent conditions among students, such as common colds, coughs, phlegm, sinusitis, and bronchitis.

We noted that the presence of air-conditioning in university classrooms was associated with lower absentee rates and improved performance, similar to what has been reported in schoolchildren occupying AC rooms [24]. Air-conditioning systems are designed to control temperature and humidity (a positive effect); however, they may also become contaminated with biological pollutants (a negative effect) if they are not judiciously maintained. A review on building investigation reports suggests significant benefits to health and equipment performance that presumably result from properly maintained HVAC systems providing consistently good thermal and ventilation control while also reducing the risk of biological contamination [25].

The average concentration of airborne dust in the NV and AC classrooms was elevated. These high concentrations were due to outdoor sources of particulate matter (PM) that may go indoors. Lee and Chang [26] revealed maximum indoor PM_10_ concentrations of 472 μg/m^3^ in classrooms, which exceeded the Hong Kong standards. The concentrations of airborne dust in this study were positively and significantly correlated with throat irritation in students from the AC rooms. 

Norback [27] and colleagues conducted a study of air quality in universities’ computer rooms and detected an average of 18 μg/m^3^ airborne dust. They also noticed that, in improved indoor air quality rooms, students experienced fewer headaches and tiredness demonstrating that poor air quality directly affects the health of occupants. The average airborne dust concentrations in primary school classrooms in Athens was 229 μg/m^3^ [28]; this result demonstrates that the concentration of indoor dust in classrooms varies from study-to-study and country-to-country. Therefore, based on world literature, our results showed higher concentrations of airborne dust in the AC rooms than in the NV rooms.

The level of airborne dust was three times greater in AC rooms than in NV rooms; this result was influenced by the type of furniture, equipment, and activities in these rooms. Some of the AC rooms with the highest dust rates were labs where students had theoretical and practical classes, and in which chemicals, soil, and rock samples were stored along with taxidermy-treated animals and animals fixed in formaldehyde. Moreover, experiments involving the release of vapors were performed in these rooms.

The NV rooms tended to have higher fungal concentration than the AC rooms. probably as a result of outdoor fungi infiltration. In addition, higher temperature and relative humidity could have contributed to the increased indoor airborne fungi levels in these rooms. The difference in I/O ratios from NV and AC rooms was statistically significant. 

Pegas and colleagues [29] studied the concentration of bacteria and fungi in three elementary schools in Lisbon, Portugal, and observed that the total bacterial and fungal colony-forming units (cfu) in all schools, both from indoor and outdoor air samples, were above the advised maximum value of 500 cfu/m^3^ defined by the Portuguese legislation.

One study revealed that the concentration of fungi and bacteria was higher in occupied than in vacant classrooms. Human occupancy in a university classroom was found to result in a significant increase in indoor airborne particulate mass, bacterial genomes, and equivalent fungal genomes. The greatest increases in particle mass and fungal equivalent genomes were observed in the largest particle sizes, which is broadly consistent with expectations of progressively increasing influence of resuspension with increasing particle size with the presence of large (>10 μm) multicellular fungal spores in indoor environments. Enhanced bacterial concentrations during occupancy were predominantly found in particles with aerodynamic diameters within the 3 μm to 5 μm range [30].

In addition, direct human contributions such as skin shedding, talking, coughing, and sneezing may play a significant, but less well-characterized role, influencing the content and character of indoor microbiological aerosols. Investigators have previously noted the significant content of desquamated human skin cells in aerosols in occupied settings and the presence of bacteria including *Staphylococcus*, *Propionibacteria*, *Corynebacteria*, and enteric bacteria, which are typically ascribed to human microflora [30,31]. A survey in three university laboratories in Italy discovered that the most frequently observed bacteria were *Staphylococcus*, *Bacillus*, and *Actinomyces* [32]. The fungi most commonly found indoors were *Alternaria* spp., *Aspergillus* spp., and *Cladosporium* spp. [30].

The presence of a small number of airborne microorganisms in indoor environments is a normal condition, however, an increase in their concentration could represent a disease risk factor [33]. Most of the studies have shown a relationship between allergic symptoms, especially respiratory, and the presence of indoor molds [10,34,35].

Several studies suggest that a high concentration of microbial air contamination, combined with other non-biological factors, could induce diverse adverse health effects such as infectious diseases, allergic and irritant responses, respiratory problems, and hypersensitivity reactions [36,37,38].

Microorganisms in indoor air originate from activities by occupants, contaminated building materials, furnishings, and outdoor air; therefore, adequate indoor air exchange and ventilation rate are commonly accepted as an essential procedure to protect occupants' health and decrease the indoor microbial charge [39].

In this study, the percentage of SBS symptoms were high in both types of studied classrooms. In a study of Finnish office worker population (*n* = 11,154), the most common symptoms of poor indoor air were irritated, stuffy, or runny nose (20%), eye itching, burning, or irritation (17%), and fatigue (16%) [40]. A study with 2365 students in Korea occupying classrooms with mechanical ventilation found that 12% had headaches and 30% fatigue [41]. The present study showed associations between greater school absenteeism and exposure to adverse building conditions such as mold, moisture, ventilation problems, and a few related system and structural problems. We did not observe a correlation between absenteeism and SBS. However, few studies have evaluated associations between school building conditions and student absenteeism.

Most studies of indoor allergens have focused on home environments. However, schools and universities may be important sites of allergen exposure for students. A significant correlation between the number of pet owners and building-related symptoms was observed in this study. Pet allergens are found in schools and universities because they are transferred to these environments on clothes, bags, and other personal items from students and staff [42]. There is strong evidence that clothing is the primary transferring mechanism and source of pet allergens [43]. A recent study suggested that, in addition to clothing, human hair may be a source of transfer and deposition of pet allergens among schoolchildren [44]. Previous studies demonstrated contamination of classrooms with cats, dogs, and house dust-mites allergens, which could cause symptoms of asthma and allergy [45,46,47]. The air quality in the school environment should not be neglected; indoor allergens exposure needs to be further investigated.

In summary, our data suggest that schools and universities can be important sites of exposure to cat and dog allergens, particularly for sensitive individuals. However, not all studies link these environments to elevated exposure levels. The number of pet owners among school or university students is one of the strongest predictors of elevated cat and dog allergen levels in these settings.

## 5. Conclusions

According to the obtained results, we conclude that the studied AC rooms show parameter values that do not comply with the standard Brazilian legislation for air quality suggesting that the performance of maintenance, housekeeping, and control of air conditioning activities affected the quality of indoor air. These parameters are directly related to public and occupational health and are excellent indicators of SBS. Appropriate methods for maintaining and cleaning classrooms could decrease dust air concentrations in these environments. Moreover, lowering occupancy and increasing breaks between classes could alleviate high CO_2_ concentrations.
